# Porcine *SOX9* Gene Expression Is Influenced by an 18bp Indel in the 5’-Untranslated Region

**DOI:** 10.1371/journal.pone.0139583

**Published:** 2015-10-02

**Authors:** Bertram Brenig, Yanyu Duan, Yuyun Xing, Nengshui Ding, Lusheng Huang, Ekkehard Schütz

**Affiliations:** 1 Institute of Veterinary Medicine, Georg-August-University, Burckhardtweg 2, D-37077, Göttingen, Germany; 2 Key Laboratory for Animal Biotechnology of Jiangxi Province and the Ministry of Agriculture of China, Jiangxi Agricultural University, 330045, Nanchang, China; Leibniz Institute for Age Research - Fritz Lipmann Institute (FLI), GERMANY

## Abstract

Sex determining region Y-box 9 (SOX9) is an important regulator of sex and skeletal development and is expressed in a variety of embryonal and adult tissues. Loss or gain of function resulting from mutations within the coding region or chromosomal aberrations of the *SOX9* locus lead to a plethora of detrimental phenotypes in humans and animals. One of these phenotypes is the so-called male-to-female or female-to-male sex-reversal which has been observed in several mammals including pig, dog, cat, goat, horse, and deer. In 38,XX sex-reversal French Large White pigs, a genome-wide association study suggested *SOX9* as the causal gene, although no functional mutations were identified in affected animals. However, besides others an 18bp indel had been detected in the 5′-untranslated region of the *SOX9* gene by comparing affected animals and controls. We have identified the same indel (Δ18) between position +247bp and +266bp downstream the transcription start site of the porcine *SOX9* gene in four other pig breeds; i.e., German Large White, Laiwu Black, Bamei, and Erhualian. These animals have been genotyped in an attempt to identify candidate genes for porcine inguinal and/or scrotal hernia. Because the 18bp segment in the wild type 5′-UTR harbours a highly conserved cAMP-response element (*CRE*) half-site, we analysed its role in *SOX9* expression *in vitro*. Competition and immunodepletion electromobility shift assays demonstrate that the *CRE* half-site is specifically recognized by CREB. Both binding of CREB to the wild type as well as the absence of the *CRE* half-site in Δ18 reduced expression efficiency in HEK293T, PK–15, and ATDC5 cells significantly. Transfection experiments of wild type and Δ18 *SOX9* promoter luciferase constructs show a significant reduction of RNA and protein levels depending on the presence or absence of the 18bp segment. Hence, the data presented here demonstrate that the 18bp indel in the porcine *SOX9* 5′-UTR is of functional importance and may therefore indeed be a causative variation in *SOX9* associated traits.

## Introduction

Sry (sex determining region Y)-box 9 (SOX9) belongs to the SoxE subgroup of Sox family proteins and is expressed during embryonal development and adult life in meso-, ecto- and endoderm derived tissues [1]. It is involved in numerous cellular processes, *e*.*g*. chrondrogenesis [2], sex determination [3], pigmentation [4], organ maintenance [1], limb development [5], and cancer [6]. In the mesoderm SOX9 is involved in chondrogenesis and skeletal development [7], male gonad development, as well as development of cardiac valves and septa as well as epithelial differentiation in the pyloric sphincter [8]. However, SOX9 does not only play an important role in embryonal development. Recent data demonstrate that it is also important in the maintenance of adult organs and expression of *SOX9* can be detected in adult stem and progenitor cells [9]. In mice it was shown that *SOX5*, *SOX9*, and *SOX13* are expressed in adult Leydig cells and may therefore contribute to steroido- and spermatogenesis in postnatal testes [10]. In rats *SOX9* expression was detected in the adult testicular cords and seminiferous tubuli suggesting a role in further germ cell differentiation [11]. *SOX9* expression was also detected in goat testis in postnatal development, however, expression levels decreased to less than 50% of the concentration measured at two months of age [12].

Despite high levels of expression in both chondrogenic tissue and gonads, *SOX9* is also transcribed to varying degrees in other tissues, including human adolescent heart, brain, kidney, muscle, colon, and cranial neural crest [13]. This suggests that SOX9 has other crucial functions not only in chondrogenesis and sex determination. For example, SOX9 supports tumor growth and invasion, regulates CEACAM1 expression in colon epithelium and plays a role in cranial neural crest development [14, 15].

Because of its wide range of interactions and functions, it is not surprising that mutations of the *SOX9* gene locus are causative for a variety of defects in humans including campomelic dysplasia with or without sex reversal [16, 17], Pierre Robin sequence [18], Cooks syndrome [19], 46,XY gonadal dysgenesis [20], 46,XX male sex reversal, and congenital generalized hypertrichosis with or without hyperplasia [21]. Male-to-female or female-to-male sex reversal has also been described in animals [22–26]. In a Sry-negative XX European roe deer three *SOX9* copies were detected leading to an incomplete male-determination. The sex reversal was presumably due to a dosage effect. In earlier studies of canine XX sex reversal *SOX9* was initially excluded as candidate gene, however, recent reports show a *SOX9* duplication resulting in an overexpression [25, 27]. In pigs female-to-male sex reversal of 38,XX animals has been described in several studies and it was shown that *SOX9* expression is elevated in XX sex-reversed or intersex gonads [23, 24]. Recently, a genome-wide association study performed in the French Large White population demonstrated that the only significantly associated SNPs clustered around the *SOX9* locus [28]. Comparative sequencing of the candidate region in affected animals and controls revealed 14 different polymorphisms. Unfortunately, all of these were located outside of the exons or splice-sites and therefore were questioned as functional candidate mutations [28]. However, at least three haplotypes were deduced that were more frequently present in the affected animals. These haplotypes included polymorphisms located in important regulatory regions as well as the 5′- and 3′-UTR of *SOX9*. One of the regulatory regions is the so-called TES (testis specific enhancer) approx. 13 kb upstream the transcription start site. It was shown in mice that within this region a highly conserved core element TESCO is bound by different transcription factors resulting in either up or down regulation of *SOX9* [3, 29]. But *SOX9* expression regulation is complex and under the control of further distant located elements. For example, eight (E1–E8) evolutionary conserved elements have been identified by comparative analysis. Five of these elements are dispersed in a region 290 kbp upstream and three up to 452 kbp downstream of *SOX9*. Two further elements were localized in the 3′-UTR of *SOX9* [30]. In recent experiments using chromosome conformation capture-on-chip analysis even more distant regulatory regions influencing *SOX9* expression have been identified [31]. These regions are located 2.46 Mb upstream as well as 1.22 Mb and 1.6 Mb downstream of *SOX9*. *SOX9* also regulates its own expression in a positive feedback mechanism by the so-called SOM, an enhancer identified in mice 70 kb upstream of *Sox9* [32]. In this respect it is noteworthy that a potential SOX9 consensus binding site [33] can be found approx. 17.8 kb upstream the porcine *SOX9* transcription start site. Besides the long-range regulation, *SOX9* expression is, of course, also regulated by promoter elements. In the murine *Sox9* promoter an interval between -193 and -73 was identified to be essential for maximal promoter activity and tissue-specific expression in mouse cell lines [34]. Subsequently, several studies reported that functional elements in this region, *i*.*e*. two functional CCAAT boxes, a cAMP-response element (*CRE*) half-site and two specificity protein 1 (SP1) binding sites within 150 bp upstream the transcription start site (TSS), regulated *SOX9* transcription in mouse and human chondrogenic cell lines and primary chondrocytes [35, 36]. In humans it was shown that *SOX9* expression is regulated by Sp1, CREB, and CBF [37, 38]. These binding sites are located in the proximal promoter upstream the transcription start site. *In vitro* mutagenesis of these sites results in a reduction of *SOX9* promoter activity. To define the regulatory network in response to different hormones and cellular signals, it is important to characterize the *cis*-regulatory elements even in the minimal promoter region. Functional elements located between -256 and +67 are important for controlling human *SOX9* transcription efficiency [35]. However, transcription elements in the interval between -73 and +251 determine the minimal transcriptional activity of the mouse *Sox9* promoter [34]. Whether additional regulatory elements are influencing *SOX9* transcription downstream the transcription start site has not been shown so far.

In this context, it is noteworthy that in the French Large White population mentioned above, an 18bp indel was detected in the 5′-UTR in three of the sequenced control animals [28]. In this contribution we report that the same 18bp indel is also present in other pig breeds, *i*.*e*. German Large White, Laiwu Black, Bamei, and Erhualian. The occurrence of the 18bp indel in such diverse pig breeds in conjunction with the results reported about the porcine 38,XX sex reversal phenotype prompted us to address the question whether this region has an influence on *SOX9* expression and might therefore at least partly explain the sex reversal. This hypothesis is further supported by the fact that the 18bp indel region harbours a *CRE* half-site which is usually recognized by the cAMP-response element binding protein CREB [39].

## Materials and Methods

### Animals

Seven Chinese domestic pig breeds (Erhualian, Laiwu Black, Bamei, Wuzhishan, Hang, Jianhexiang White, Tibetan) and different Western commercial pig breeds (Pietrian, German Landrace, German Large White, Red Duroc) were investigated. Blood samples were drawn by veterinarians as part of routine diagnostic procedures (parentage control, epidemiological testing) with informed owner consent, therefore the study was exempt of ethical approval according to the German regulations. A total of 938 animals were used for the identification of polymorphisms in the *SOX9* gene. The complete *SOX9* gene including 2 kb up- and downstream sequences was analysed.

### PCR fragment length polymorphism (PFLP) analysis

PFLP analysis was used to screen all samples using the following PCR primers: forward 5'-GCCAGTTTTACCCCCAGGA–3' and reverse 5'-AGCGGCTCCCGGGAA GCCT–3'. The amplicon has a length of 189bp and 207bp for the Δ18 and wild type alleles, respectively. PCR was performed in a final 20 μL reaction mix contained 40 ng DNA, 1 × PCR buffer, 2.0 mM MgCl_2_, 0.2 mM of each dNTP, 10% DMSO, 10 μmol of each primer and 1 unit of ExTaq DNA polymerase (Takara). The PCR profile was as follows: 3 min at 94°C; 35 cycles of 30 s at 94°C and 1 min at 68°C; and a final extension of 7 min at 72°C. PCR products were evaluated by electrophoresis on 2% agarose gels.

### Tissue culture

The cell lines used for the experiments were selected based on their reported transfection efficiencies and level of *SOX9* expression. Nuclear extract from ESK–4 cells (female embryonal porcine kidney) were used for the immunodepletion EMSA in order to keep within the autologous porcine system. In addition it was shown previously that *SOX9* is expressed in the embryonal kidney and therefore the presence of essential regulatory factors was assumed [40]. No data on *SOX9* expression in PK–15 are available. Expression of *SOX9* has been shown in ATDC5 [41]. In HEK293T SOX9 was not detectable using antibody NBP1-85551 (Novus Biologicals). However, due to a lower transfection efficiency of porcine epithelial PK–15 and mouse pre-chondrogenic ATDC5 cell lines, we also used human kidney HEK293T, which is known to have a higher transfection efficiency [41–44].

HEK293T, PK–15, and ATDC5 cells were cultured in Dulbecco’s modified Eagle’s medium supplemented with 10% fetal bovine serum and 2 mM glutamine at 37°C in a humidified atmosphere containing 5% CO_2_. Porcine kidney ESK–4 cells were purchased from the European Collection of Cell Cultures (ECACC) and cultured according to the manufacturer’s recommended protocols.

### Affinity purification of CREB

DNA-binding affinity was analysed as described [45] using three oligonucleotides containing either the *CRE* half-site (WT: 5′-CGCTGCAGCCGAGTGACGCGCCAG GCTTCC–3′), a mutated *CRE* half-site_mut_ (MT: 5′-CGCTGCAGCCGAGCTCTACGC CAGGCTTCC–3′) or the consensus *CRE* binding site (CRE: 5′-AGAGGATTGCCT GACGTCAGAGAGCTAG–3′). HEK293T cells were transfected using HA-tag CREB-expression plasmid (pcDNA4-HA-CREB, see below) and nuclear and cytoplasmic extracts were prepared after 32 h. Extracts were incubated with the biotinylated probes (WT, MUT, CRE) and Streptavidin MagneSphere Paramagnetic particles (Promega) for 2 h at room temperature. The reactions were separated using a MagneSphere Magnetic Separation Stand (Promega) and washed according to the manufacturer’s instructions. Eluted extracts were separated on SDS/PAGE and analysed by Western blotting using a phospho-CREB antibody (Millipore).

### Electromobility shift assay (EMSA)

Nuclear and cytoplasmic extracts were prepared from sub-confluent ESK–4 cells by NE-PER Nuclear and Cytoplasmic Extraction Kit (PIERCE) according to the manufacturer’s instructions. The DNA-protein interaction was studied by EMSA following the instructions of Chemiluminescent Nucleic Acid Detection Module (PIERCE) with minor modifications as previously described [46]. Briefly, binding reactions included 2 μL 10 x binding buffer (100 mM Tris, 500 mM KCl, 10 mM DTT; pH 7.5), 5% BSA, 0.5 mM EDTA, 5% glycerol, 1 mM MgCl_2_, 100 ng poly-dAdT, 0.1% NP–40 and 4–4.5 μg nuclear and cytoplasmic proteins. Binding reactions were pre-incubated for 20 min on ice followed by 20 min at room temperature after adding 20 fmol biotin-end labelled double strand oligonucleotide probe (wild type: 5′-gcgccttcctaaaagctcgccgcTGCAGCCGAGTGACGCGCcaggcttcccgggagcc–3′; Δ18: gcgccttcctaaaagctcgccgccaggcttcccgggagcc–3′). Finally, the mixtures were loaded onto 7% acrylamide–0.5x Tris-borate-EDTA gel (acrylamide/bisacrylamide = 37.5:1). For competition analysis, 2–5 pmol unlabelled competitors, *i*.*e*. the 18-bp fragment, were added to the binding reactions. For interrogation of the binding transcription factor, 3 μL phospho-CREB antibody (Millipore) or E2F4 antibody (Millipore) was pre-incubated with nuclear and cytoplasmic proteins on ice for 2 h to get the maximal signal. The mix was added to the above binding reactions for 20 min on ice prior to the addition of the labelled DNA probe.

### Plasmid constructs

To analyse the effect on expression of CREB binding to the wild type 5′-UTR pcDNA4-HA-CREB, pGL4.20-WT (wild type) and pGL4.20-Δ18 (mutant) were constructed. Full-length human CREB cDNA (Sino Biological Inc.) was subcloned into the pcDNA4-HA expression vector. pGL4.20-WT and pGL4.20-Δ18 was generated by subcloning the porcine *SOX9* promoter between position -100 and +399 (TSS = +1) into the *Kpn*I/*Hin*dIII sites of the pGL4.20 vector. The promoter was obtained by artificial gene synthesis (Sangon).

To determine the effect of Δ18 on translation, two plasmids (psiCHECK-2-WT-TTG and psiCHECK-2-Δ18-TTG) were constructed by inserting the 5’-UTR plus the start codon of *SOX9* into an *Nhe*I site upstream the *Renilla* luciferase gene. Before inserting the DNA fragments into the psiCHECK–2 vector, the start codon of the *Renilla* luciferase was mutated to TTG by PCR-driven overlap extension. By this way, the *Renilla* luciferase translation would be driven by the start codon of *SOX9*. The 5'-UTR of *SOX9* was PCR amplified using the primers: forward 5'-TTTTGCTAG CAAGAGCCCCTGGGCTGGGAGTTGG–3' and reverse 5'-TTTTGCTAGCATCAC GCAGGCCCGGGGCAGGGGAC–3'. Amplified products were cloned and ligated into the *Nhe*I site directly preceding the *Renilla* luciferase gene in the plasmid psiCHECK-2-TTG. The psiCHECK2-TTG vectors are ideal for examining the effect of 5’-UTRs on gene expression as described [47, 48]. By fusing the 5’-UTR of interest to the TTG mutated *Renilla* luciferase gene, luciferase activity can be used as a marker for 5’-UTR regulation.

### Luciferase reporter assays

Cells were seeded at 2 x 10^5^ cells per well in 24-well dishes. When the cells were 80% confluent, the luciferase constructs were transfected using Lipofectamine^TM^ 2000 (Invitrogen) according to the manufacturer’s instructions. Thirty-six hours after transfection, cells were harvested and assayed for luciferase activity using a dual-luciferase reporter assay kit (Promega) following the manufacturer’s manual. Results are given as relative light units (RLU) that is Firefly luciferase value/Renilla luciferase value. The average RLU of cells co-transfected with pGL4.20 and pRL-TK (*Renilla* luciferase control vector, Promega) was considered to be 1. In each experiment 0.2 μg of pGL4.20, pGL4.20-WT, or pGL4.20-Δ18 was added together with 50 ng pRL-TK without or with 0.8 μg pcDNA4-HA-CREB. The optimal amount and ratio of reporter and control plasmids have been determined in preliminary experiments. The amount of pRL-TK was adjusted according to previous reports [49–51].

Student's t-test was used to calculate significance (* p<0.05, ** p<0.01, *** p<0.001). All experiments were performed in triplicate.

### Real-time quantitative PCR assays

Total RNA was extracted with the TRIZOL Reagent (Invitrogen) according to the manufacturer’s instructions. 6 μg of RNA was transcribed into first-strand cDNA using Superscript First-Strand Synthesis System for RT-qPCR (Takara). The relative expression levels were analysed using Brilliant II FAST SYBR QPCR Master Mix (Stratagene) in an MX3000P system (ABgene) according to the manufacturer’s instructions. The following primers were used: Renilla 1, 5′-CTGATCTGATCGGAA TGGGT–3′ and 5′-GACGATGGCCTTGATCTTGT–3′; ß-actin 1, 5′-AAGGAGAAG CTGTGCTACGTCG–3′ and 5′-TGAAGGTAGTTTCGTGGATGCC–3′.

### Statistical analysis

Putative transcription factor binding sites within Δ18 were analysed with MatInspector (http://www.genomatrix.de/matinspector) using default settings. The DNA sequence between the transcription start site and the start codon was used as input. The promoter alignment analysis of evolutionary relationship among pig and other vertebrates was performed using ClustalW2 (http://www.ebi.ac.uk/). Expression data were analysed using GraphPad Prism^TM^ 5.0 for Macs (GraphPad Software, San Diego, CA).

All transfection experiments were performed in at least three independent experiments using three biological replicates and standard errors (SEM) were calculated using Microsoft Excel for Mac 2011 (version 14.5.4).

## Results

### CREB binds to the *CRE* half-site within the 5'-UTR of the porcine *SOX9* gene and influences expression

In an analysis of 938 animals of different commercial and Chinese pig breeds 12 polymorphisms (SNP) were identified within in the 5′-UTR (2/12), introns (5/12), and 3′-UTR (5/12) of *SOX9* (Table 1). In addition an 18bp indel (Δ18) abolishing an interval between +247bp and +266bp downstream the TSS in four of the analysed pig breeds, *i*.*e*. German Large White, Laiwu Black, Bamei, and Erhualian, was identified (Table 2). As mentioned above we previously studied Δ18 in 545 animals of the commercial breeds in an attempt to identify an association with inguinal and scrotal hernia. However, no significant association could be detected using transmission disequilibrium test (p = 0.075) [52, 53]. All SNPs including Δ18 have been already deposited either with dbSNP or described previously as mentioned in the introduction [28].

**Table 1:** Polymorphisms detected in the porcine *SOX9* gene.

Position^a)^	location	dbSNP
g.66309C>T	5′-UTR	rs81219813
g.66392G>T	5′-UTR	
g.66420_66437del	5′-UTR	
g.67588G>A	intron 1	
g.67832T>A	intron 1	rs196959349
g.68580C>G	intron 2	rs81219816
g.68654G>T	intron 2	rs81219817
g.68761C>T	intron 2	rs81219818
g.70597T>G	3′-UTR	rs327718481
g.71244T>C	3′-UTR	rs324747068
g.71842C>T	3′-UTR	rs322934388
g.71902T>G	3′-UTR	rs320099199
g.71935A>G	3′-UTR	rs337315481

^a)^ Positions according to GenBank accession number AC157866.2; variants according to HGVS http://www.hgvs.org/mutnomen/ (last accessed on May 29th, 2015)

**Table 2:** Genotype distribution of the 18-bp variant in different commercial breeds, seven Chinese domestic breeds and three Western commercial breeds.

Breed	n	Genotype		
		wt/wt^b)^	Δ18/wt	Δ18/Δ18^c)^
Different commercial breeds^a)^	665	388	235	42
Erhualian	59	58	1	0
Laiwu Black	37	25	7	5
Bamei	35	23	7	5
Wuzhishan	29	29	0	0
Hang	43	43	0	0
Jianhexiang White	28	28	0	0
Tibetan (Milinzang)	42	42	0	0

^a)^ Commercial breeds included Pietrain, German Landrace, Large White, Red Duroc;

^b)^ pigs harbouring the wild type allele (homozygous);

^c)^ Δ18: pigs harbouring the 18-bp deletion allele (homozygous).

**Fig 1:**
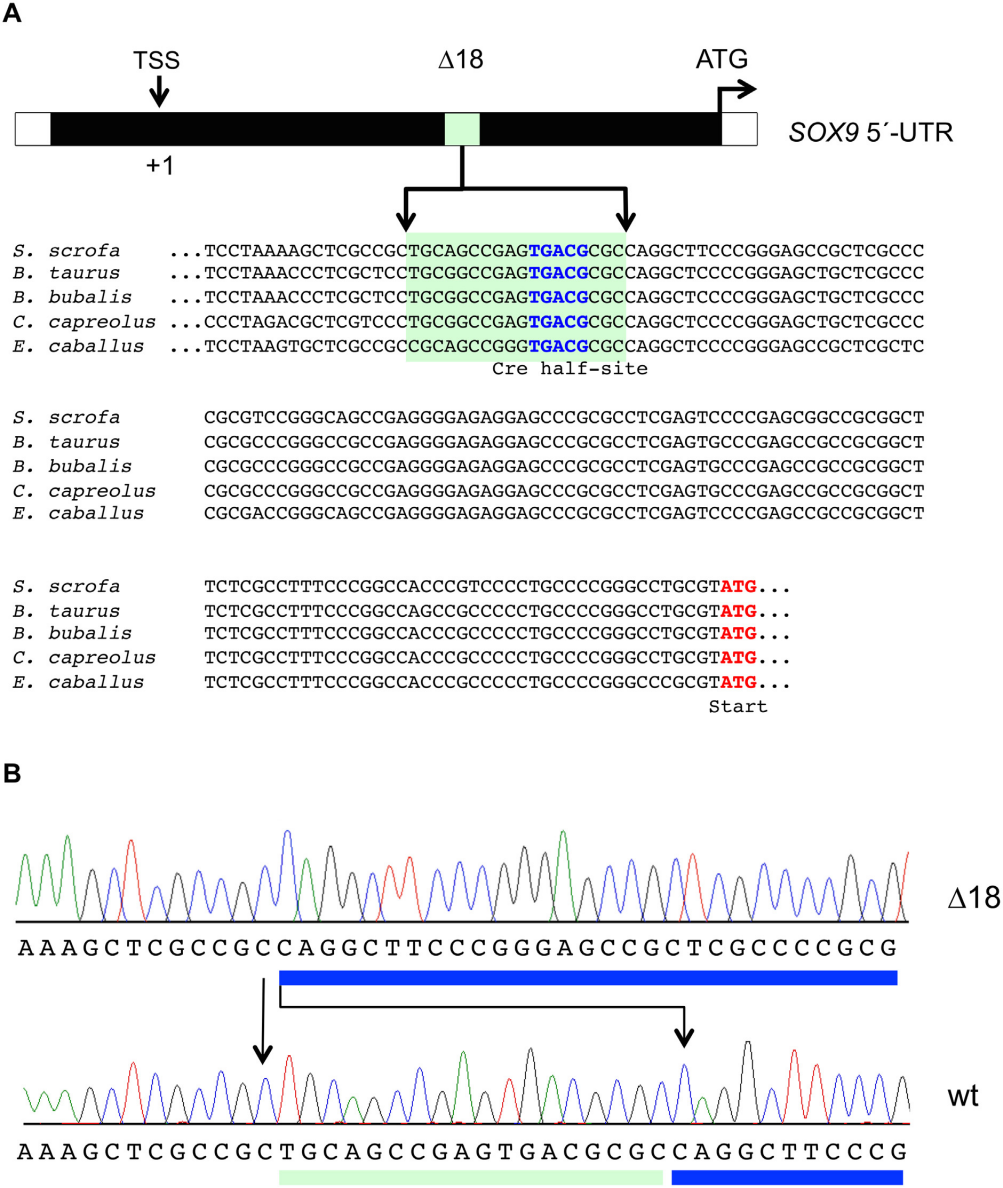
Organization and location of the proximal promoter of the porcine *SOX9* gene. The 18bp sequence and the *CRE* half-site are indicated. The sequence alignment in the lower panel shows conserved regions between pig, cattle, buffalo, roe deer, and horse. (B) Sequence alignment of the wild type and 18bp indel (Δ18) region.

**Fig 2:**
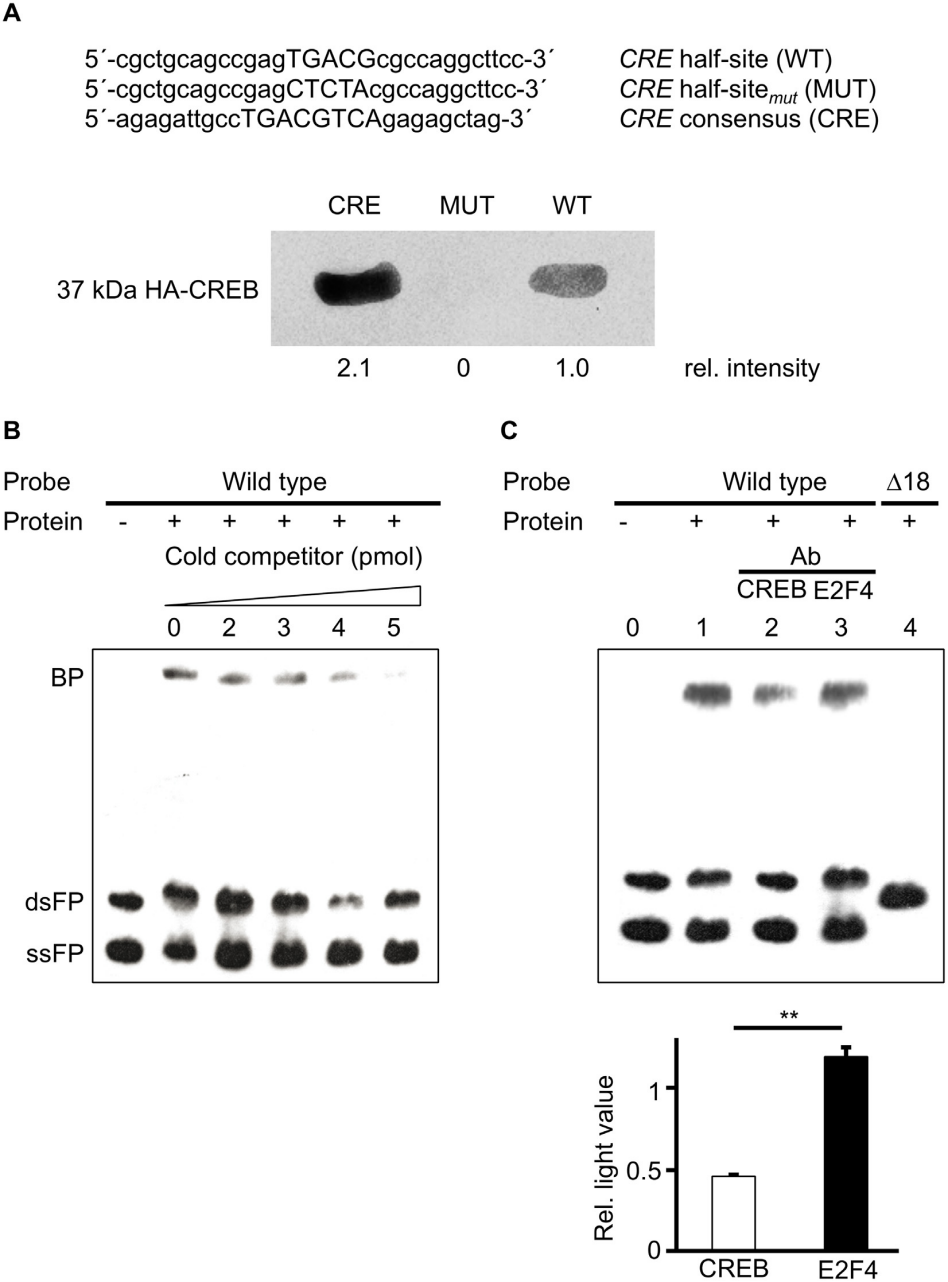
CREB binds the *CRE* half-site in the 18bp region. (A) Biotinylated oligonucleotides WT, MUT, and CRE were incubated with cell extracts of HEK293T cells transfected with a CREB expression plasmid. Bound protein was purified using Streptavidin MagneSphere Paramagnetic particles and a MagneSphere Magnetic Separation Stand (Promega), separated on an SDS/PAGE and Western blotted. Western blots were incubated with an HA-CREB antibody. Intensities of bands were densitometrically measured using ImageJ software (http://imagej.nih.gov/ij/index.html). (B) A constant amount of nuclear and cytoplasmic proteins of ESK–4 cells and labelled probe (WT) were incubated with increasing amounts of homologous unlabelled competitor. DsFP, double stranded free probe; ssFP: single stranded free probe; BP, bound protein-DNA complexes. (C) Immunodepletion assays were performed using anti-phospho-CREB and anti-E2F4 antibodies. Density of the resulting bands was determined using ImageJ software.

DNA sequence comparison between pig, cattle, buffalo, roe deer, and horse, demonstrates that the Δ18 region is highly conserved (Fig 1) and harbours three potential transcription factor binding sites with varying degrees of similarity to the respective consensus motives (Table 3). Due to the highly conserved sites and potential transcription factor binding sites, we hypothesized that this region might be important for *SOX9* expression. To test CREB binding to the 18bp region *in vivo*, HEK293T cells were transfected with the CREB-expression plasmid (pcDNA4A-HA-CREB) and nuclear and cytoplasmic extracts were incubated with oligonucleotides harbouring the 18bp region (WT) and a *CRE* consensus sequence (CRE). As control an oligonucleotide homologous to the 18bp region with a mutated *CRE* half-site (MUT) was used. As shown in Fig 2A CREB binds to the WT and CRE oligonucleotides, whereas no binding was detected to the mutated control oligonucleotide. Specificity of CREB binding was analysed with competition and immunodepletion EMSAs. Nuclear and cytoplasmic extracts were prepared from ESK–4 cells and incubated with the labelled wild type DNA fragment (Fig 2B). As shown in Fig 2B, a specific band (BP) was detectable and diminished with increasing concentrations of cold unlabelled double strand homologous competitor. To exclude other factors which could bind to this region, we used antibodies directed against another potential transcription factor listed in Table 3, *i*.*e*. E2F, pre-incubated the cell extracts with the antibodies and then performed an immunodepletion EMSA as described elsewhere [54]. With the interference analysis we expected that due to binding of the antibody less transcription factor would be available to bind the labelled oligonucleotide and therefore would result in a reduced band intensity. Two antibodies were used, directed against CREB and E2F4. Fig 2C shows that when a polyclonal CREB antibody (anti-phospho-CREB) was added to the binding reaction the intensity of the protein-DNA complex was significantly reduced (lane 2) whereas an anti-E2F4 antibody did not influence the band intensity (lane 3). No binding was detected with the Δ18 fragment (lane 4). The band intensities were densitometrically quantified and differed significantly between lane 2 and 3 (Fig 2C lower panel).

**Table 3:** Putative transcription factor binding sites within the 5'-UTR between positions 66,414 to 66,446 of the porcine *SOX9* gene.

TF	Position^a)^		Strand	Similarity	Sequence motif (5′-3′)^b)^
					GCTCGCCGCTGCAGCCGAGTGACGCGCCAGGCT ^c)^
CREB	66,433	66,437	+	100%	NNNNNNNNNNNNNNNNNNNTGACGNNNNNNNNN
E2F^d)^	66,437	66,440	-	50%	NNNNNNNNNNNNNNNNNNtttcCGCGNNNNNNN
E2F^d)^	66,436	66,440	+	62.5%	NNNNNNNNNNNNNNNNNNNtttCSCGCNNNNNN
ZF5	66,436	66,440	+	75%	NNNNNNNNNNNNNNNNNNNNNgSGCGCgRNNNN

^a)^ Nucleotide positions refer to accession number AC_157866.2;

^b)^ IUPAC-IUB ambiguity code: S = G or C, R = A or G, N = any, lower case letters indicate mismatching nucleotides compared to the transcription factor consensus motif;

^c)^ DNA sequence corresponds to porcine *SOX9* gene between positions 66,414 to 66,446. The Δ18 region is underlined;

^d)^ The consensus motif corresponds to the binding site of all E2F family members.

**Fig 3:**
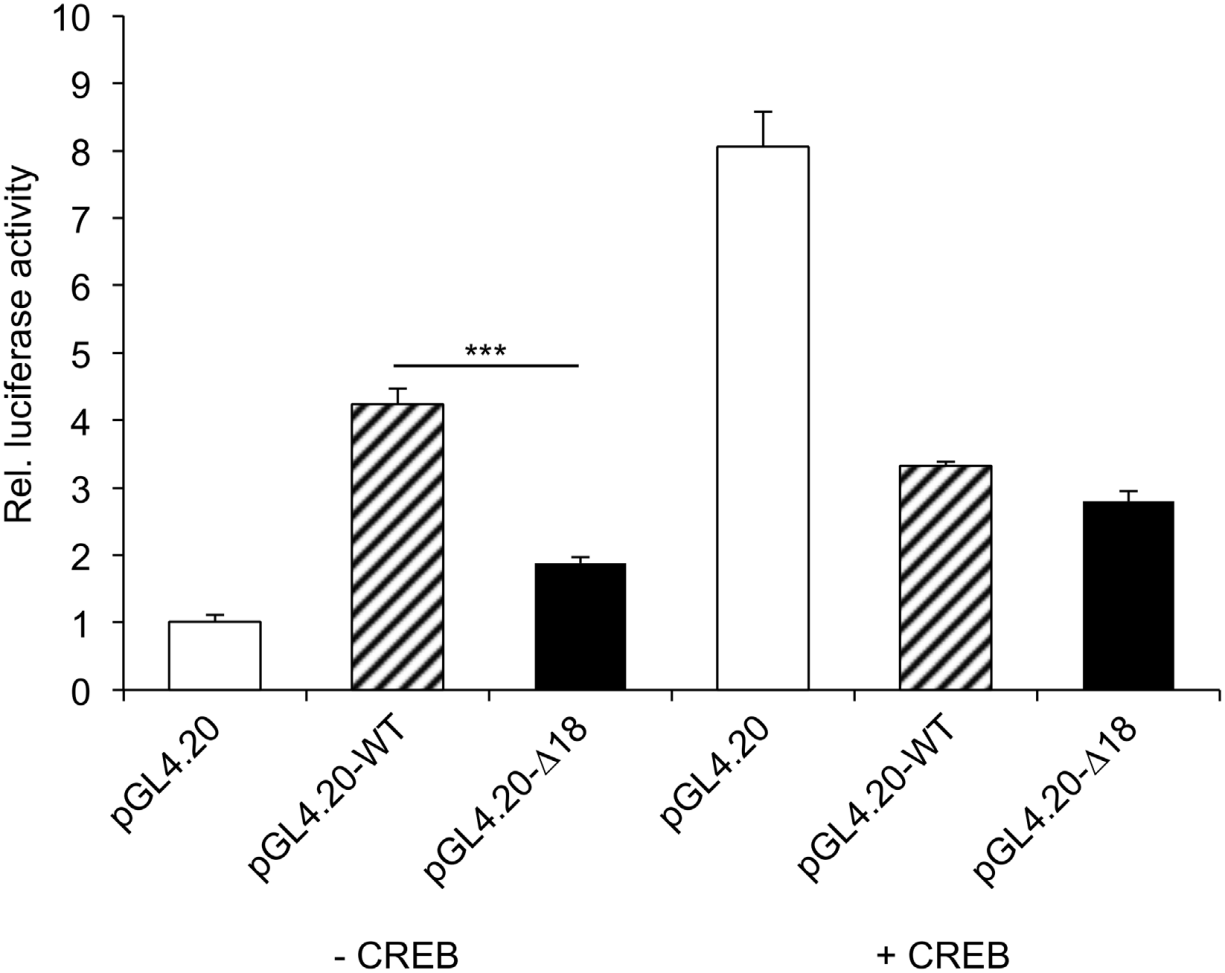
Binding of CREB to the wild type 5′-UTR and Δ18 results in reduced expression. HEK293T cells were transiently co-transfected with pGL4.20-WT, pGL4.20-Δ18 with or without pcDNA4A-HA-*CREB* over-expression vector. Relative luciferase activity was measured after 32 h using dual luciferase reporter assays. The results are given as relative light units (RLU). The average RLU of cells co-transfected with pGL4.20, pRL Renilla luciferase control reporter vector, and pcDNA4A-HA was considered to be 1 (error bars indicate standard deviations).

To analyse whether CREB binding and the Δ18 variant in the context of the 5'-UTR have regulatory effects on *SOX9* expression the wild type and the Δ18 5′-UTR were cloned into the pGL4.20 vector and used for transfection of HEK293T cells with or without a CREB expression plasmid. As shown in Fig 3 (left panel, -CREB) the wild type 5′-UTR resulted in a significantly higher activity than the Δ18 fragment in the absence of CREB. When CREB was co-expressed (Fig 3 right panel, +CREB) the activity of the wild type 5´-UTR was reduced to the level of the Δ18 fragment and both fragments resulted in a significant lower expression than the CREB induced expression of the control vector.

### Effects of the variable *SOX9* 5'-UTR on transcription and translation in different cell types

**Fig 4:**
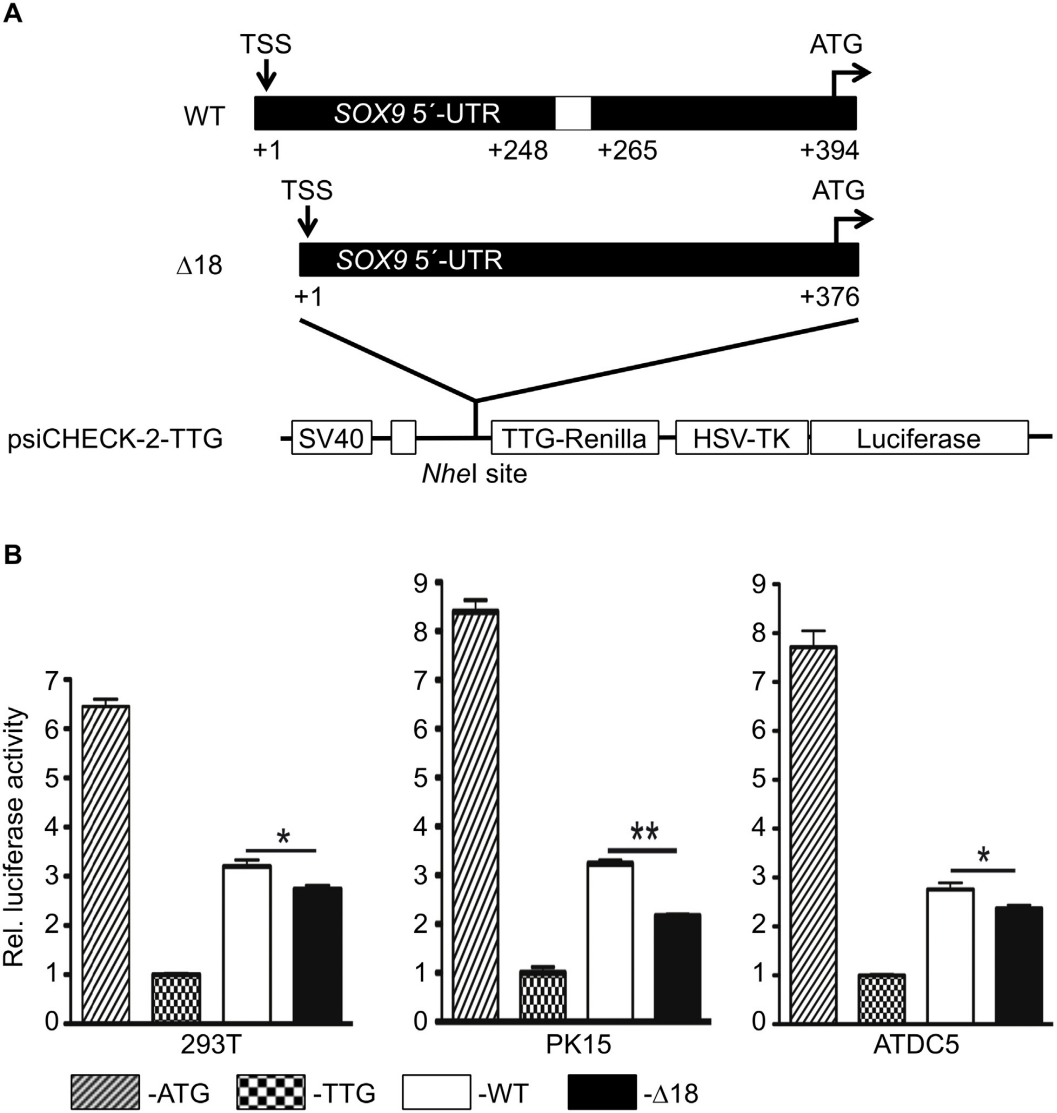
Δ18 reduces translation efficiency. (A) Schematic diagram showing the luciferase plasmids used for translation efficiency analysis. The start codon of *Renilla* was mutated to TTG. Two plasmids (psiCHECK-2-WT-TTG and –Δ18-TTG) were constructed by inserting the 5’-UTR plus the start code of SOX9 into upstream of the mutated *Renilla* luciferase gene. By this way, the *Renilla* luciferase expression would be driven by the primary initiation codon of SOX9. (B) The deletion decreases the translation efficiency of porcine *SOX9*. The four different plasmids were transfected into HEK293T, PK–15 and ATDC5 cells. After 24 h, both Firefly and Renilla luciferase values were measured. The ratio of Renilla and Firefly luciferase value were used as RLU. The average RLU for psiCHECK-2-TTG was considered to be 1.

Three expression vectors were constructed, *i*.*e*. psiCHECK-2-TTG, -WT-TTG and Δ18-TTG and transfected into HEK293T, PK–15, and ATDC5 cells. As shown in Fig 4A the *Renilla* luciferase start codon was mutated to TTG (psiCHECK-2-TTG) to abolish translation from this site. As the 5'-UTR of porcine *SOX9* with a length of 394 bp does not contain multiple open reading frames or other upstream AUGs except the natural start codon, we cloned the two different variants of the *SOX9* 5'-UTR into the *Nhe*I cloning site of the psiCHECK-2-TTG vector to generate psiCHECK-2-WT-TTG and psiCHECK-2-Δ18-TTG. In these constructs translation will be initiated from the original *SOX9* start codon -12 bp upstream with the addition of 4 amino acids at the N-terminus of the *Renilla* luciferase. Although the N-terminus of the luciferase is important for the stability of the enzyme [55], it has been shown that the addition of amino acids, *e*.*g*. Myc epitope or His-tag, does not influence the activity [56, 57].

**Fig 5:**
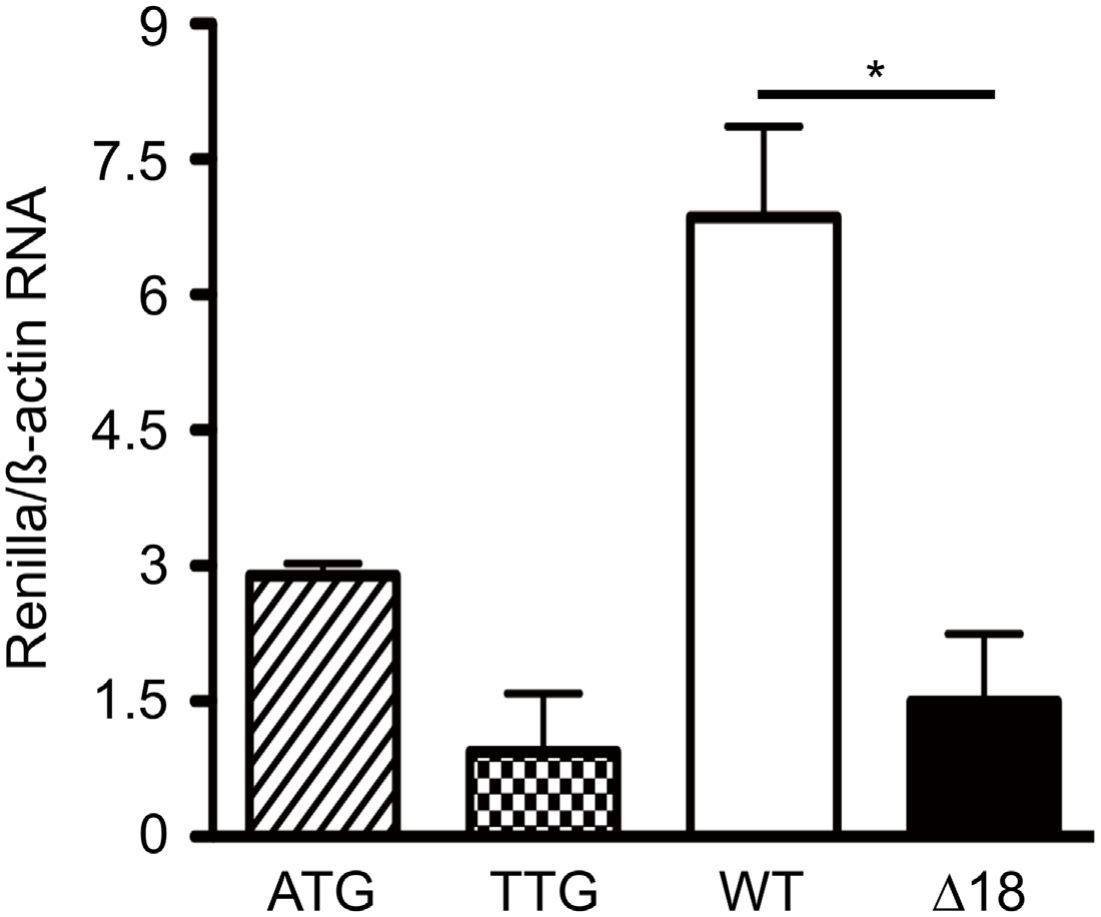
Determination of wild type and Δ18 5′-UTR transcription regulation. RT-qPCR was performed for quantifying *Renilla* mRNA contents. The content of *Renilla* was normalized to ß-actin as internal control and the relative luciferase mRNA for psiCHECK-2-TTG was fixed as 1.

In all three transfected cell types the luciferase activity was almost reduced to background levels compared to the intact expression vector demonstrating the effect of the mutated *Renilla* start codon (Fig 4B). *Renilla* luciferase activity of psiCHECK-2-TTG was then used to assess the effect of the 5’-UTR on translation. Both constructs harbouring the wild type (WT) or Δ18 were used for transfection of HEK293T, PK–15, and ATDC5 cells. In all three cells types, the Δ18 showed a significant lower luciferase activity compared to the wild type 5′-UTR construct. In addition, *Renilla* transcripts were analysed by qRT-PCR. As shown in Fig 5 the wild type 5′-UTR resulted in a 3-fold higher RNA concentration compared to Δ18.

## Discussion

In the present study we characterized the effects of an 18bp deletion within the 5’-UTR of porcine *SOX9* on transcription and translation. A sequence comparison of the 18bp region between pig and other species showed several conserved nucleotide positions including a *CRE* half-site. To determine whether the *CRE* half-site was recognized by CREB, we performed affinity purification, competition and immunodepletion mobility shift assays and were able to show binding. CREB did functionally bind to the *CRE* half-site (CGTCA/TGACG), albeit with reduced binding affinity compared to the full palindrome (TGACGTCA) [58]. CREB seems to be an important transcription factor in the regulation of bone development by activating *Sox9* in mouse. Overexpression of *Creb* enhanced the action of *Sox9* in the PKA pathway in BMP-2-induced osteochondrogenic differentiation [59]. Potent dominant negative *Creb* transgenic mice showed short-limbed dwarfism along with a defect in chondrocyte proliferation and a delay in chondrocyte hypertrophy in endochondral bone formation [60]. Other sites could also interfere with the expression, for example, an additional conserved *CRE* half-site at position -147 in the human *SOX9* promoter [37]. However, CREB binding in the porcine *SOX9* gene 5′-UTR seems to down-regulate expression as shown in Fig 3. Interestingly, CREB co-expression clearly up-regulated the promoterless control vector pGL4.20. Although it has been reported that pRL-TK used as normalizer is up-regulated by different transcription factors, e.g. GATA transcription factors [49], 12S E1A [61], SP1 [51], Nurr1 [50], and dexamethasone [62], there are no data available that CREB influences pRL-TK expression per se. However, as it is known that the TK promoter is influenced by CREB binding [63], we also expected that pRL-TK will be up-regulated and therefore took this into account when normalizing the expression levels. Hence, the increased pGL4.20 expression in fact seems to be due to a CREB dependent induction. Although this was an unexpected and so far unknown effect of CREB on pGL4.20 expression, promotorless luciferase reporter vectors pGL3 and pGL4 have been reported to be at least responsive to steroid hormones [64].

Inhibition of expression by CREB has been shown for other genes, *e*.*g*. MuSK, AP–2α, and PPAR-γ [65–67]. Truncations or mutations in UTRs often defeat fine regulation, cause impaired protein synthesis and finally associate with various diseases or disease susceptibility in humans [68]. Two studies showed that microRNA–145 directly repressed *SOX9* expression by binding a unique site in the 3’-UTR resulting in a profound change in the human chondrocyte phenotype [69, 70]. It is also known that a stable secondary structure, multiple ORFs, and uAUGs in the 5’-UTR largely determine translation efficiency [71]. However, the 5’-UTR of the porcine *SOX9* has only one ORF and hence the downregulation of *SOX9* expression seems to be due only to either CREB binding or the presence of Δ18. CREB binding resulted in a reduction of *SOX9* transcription as shown in Fig 3, whereas Δ18 could have an effect on either RNA stability or RNA degradation. A comparative *in silico* analysis of the secondary structure formation of the wild type and Δ18 5′-UTR showed that wild type RNA had a lower ΔG (-170.77) than Δ18 (-161.78) (data not shown).

Although regulation of *SOX9* expression is complex and the role of a single site must be interpreted with caution, our results are in agreement with the findings that the wild type 5′-UTR was identified more often in 38,XX sex-reversal pigs and *SOX9* is over-expressed in XX intersex gonads [23, 28]. As shown in Figs 4 and 5 the presence of the 18bp region resulted in a significant increase of promoter activity compared to Δ18. Only, when CREB was overexpressed and bound to its binding site a reduction of expression was observed (Fig 3). From these findings different scenarios for the development of a 38,XX sex-reversal phenotype can be hypothesized. In the presence of the wild type genotype a sex reversal phenotype could result from a low CREB expression and a more stable *SOX9* RNA secondary structure leading to high SOX9 concentrations and consequently to a male phenotype. On the other hand, if CREB expression is high enough to down-regulate *SOX9* expression, no sex reversal will be induced. In the presence of the Δ18 genotype, where *SOX9* expression was low independent of CREB and the RNA secondary structure was less stable, a normal sex development would be expected for 38,XX individuals.

Hence, in further experiments it will be interesting to analyse the expression of CREB in controls and 38,XX sex-reversal gonads.

## Conclusion

We have shown that an 18bp deletion located +248 bp downstream the TSS in the 5’-UTR of the porcine *SOX9* gene harbours a functional *CRE* half-site. The deletion segregates in several pig breeds. The 18bp region is essential for transcription and translation efficiency of porcine *SOX9* and interacts with CREB. CREB binding to the wild type or presence of the 18bp deletion results in a significant reduction of *SOX9* transcription and translation.
